# MRI evaluation of the role of No-Reflow in left ventricular remodeling after Acute Myocardial Infarction (AMI)

**DOI:** 10.1186/1532-429X-11-S1-P119

**Published:** 2009-01-28

**Authors:** Carlo Liguori, Luigi Natale, Agostino Meduri, Antonio Bernardini, Antonella Lombardo, Lorenzo Bonomo

**Affiliations:** 1 Università Cattolica del Sacro Cuore – Policlinico A. Gemelli, Rome, Italy; 2 "G. Mazzini" Hospital – ASL Teramo, Teramo, Italy

**Keywords:** Acute Myocardial Infarction, Acute Myocardial Infarction, Myocardial Viability, Left Ventricular Remodel, Negative Prognostic Factor

## Purpose

LV remodeling represents the most important negative prognostic factor after myocardial infarction. Aim of the study is to assess by MRI the role of edema, no-reflow and myocardial viability in the remodeling process.

## Materials and methods

32 pts with AMI and primary PCI (25 LAD, 3 CX, 4 RCA; 26: TIMI 3, 6: TIMI 2) were studied by MRI to measure end-diastolic (EDV) and end-systolic (ESV) volumes immediately and 1-month later. A >20% increase of EDV and/or ESV was considered indicative of remodeling. Triple IR-FSE for edema evaluation, steady-state free precession cine (FIESTA) for contractile function, fast-gradient echo train (FGRET) for first-pass perfusion study and IR-prep fast GRE for delayed enhancement assessment were obtained. A score for edema, no-reflow and hyperenhancement was calculated in each segment (17-segments LV model) based on number of segments and transmural extension (75%).

## Results

14 pts showed remodeling. EDV and ESV increased from 106 ± 30 ml to 153 ± 36 ml and from 60 ± 17 ml to 91 ± 23 ml in pts with remodeling. No-reflow was detected in 26 pts (81%). The scores for edema, no-reflow and hyperenhancement were 4.0 ± 1.6, 2.4 ± 1.1, 3.3 ± 1.6 respectively in pts with remodeling and 2.9 ± 2.2 (p:NS), 1.4 ± 0.9 (p:0.04), 2.6 ± 1.7 (p:NS) respectively in pts without remodeling.

## Conclusion

First-pass MRI detects an high incidence of no-reflow after PCI. Its extension was more significantly related to remodeling if compared to edema and necrotic myocardium.

No-reflow assessed by first pass imaging seems to be a stronger predictor of LV remodeling, compared to infarct size, in AMI.

